# Effect of Passive Ultrasonic Irrigation over Organic Tissue of Simulated Internal Root Resorption

**DOI:** 10.1155/2021/3130813

**Published:** 2021-10-29

**Authors:** Amjad Abu Hasna, Jaiane Bandoli Monteiro, Ricardo Toledo Abreu, Wanessa Camillo, Amanda Guedes Nogueira Matuda, Luciane Dias de Oliveira, Cesar Rogério Pucci, Cláudio Antonio Talge Carvalho

**Affiliations:** ^1^Department of Restorative Dentistry, Endodontics Division, Institute of Science and Technology, São Paulo State University (ICT-Unesp), São José Dos Campos, Brazil; ^2^Department of Dental Materials and Prosthodontics, Institute of Science and Technology, São Paulo State University (ICT-Unesp), São José Dos Campos, Brazil; ^3^Department of Restorative Dentistry, Institute of Science and Technology, São Paulo State University (ICT-Unesp), São José Dos Campos, Brazil; ^4^Department of Biosciences and Oral Diagnosis, Institute of Science and Technology, São Paulo State University (ICT-Unesp), São José Dos Campos, Brazil

## Abstract

This study aimed to evaluate the efficacy of passive ultrasonic irrigation (PUI) on dissolving the organic tissue inside simulated internal root resorption (IRR) using sodium hypochlorite (NaOCl) or chlorhexidine (CHX). A total of 40 human lower premolars were collected based on dimensional and morphological similarities. The roots were embedded in cylinders (3 cm diameter; 2.5 cm height) of self-cured acrylic resin, and then an IRR was simulated. The specimens were divided into 4 groups (*n* = 10) according to irrigation protocols: group 1: CHX + PUI; group 2: CHX; group 3: NaOCl + PUI; group 4: NaOCl. The total irrigation time was 150 s at a flow rate of 5 mL/min. A tissue mass of porcine palatine mucosa was used to simulate the organic tissue, it was weighed before and after the irrigation using an analytic balance, and the difference between both readings was calculated and transferred to percentage values. Data were submitted to statistical analysis using two-way ANOVA (factors: irrigant type and with/without PUI) and Tukey's test for multiple comparisons among the experimental groups (*α* = 0.05). There was a significant difference in both factors (irrigant: *p*=0.04; PUI: *p* ≤ 0.001). The groups that used PUI were more effective in dissolving the organic tissue of the IRR simulation than the groups without PUI. PUI is more effective than the syringe and needle irrigation in organic tissue dissolution.

## 1. Introduction

The root resorption is a hard tissue loss activated by odontoclasts [1] because of a mechanical injury such as trauma, surgical procedures, and excessive pressure of an impacted tooth or a chemical irritation by hydrogen peroxide or other irritating agents [2]. It may be internal root resorption (IRR) resulted from infected or inflamed pulp tissue or external root resorption (ERR) resulted from periodontal infection [3] where the clastic precursor cells are predominantly recruited through the blood vessels.

The control of the IRR process depends fundamentally on cutting blood supply to the resorbing tissues through root canal treatment (RCT) which remains the only choice of management for such conditions when teeth are considered restorable and have a favorable prognosis [4].

Sodium hypochlorite (NaOCl) is capable of dissolving the organic material during RCT [5], and it has a wide antimicrobial action, detoxifies endotoxins, and reduces matrix metalloproteinase (MMP) production [6–8]. However, weak evidence is found in the literature about chlorhexidine (CHX) dissolving capacity [9]; still, it has a wide antimicrobial action [10]. Otherwise, both can reach untouched areas that the mechanical instrumentation cannot [11]. This may be related to many factors including the endodontic instrument taper, mechanical properties [12, 13], and the preliminary diagnosis [14–16].

Syringes and needles, gutta percha points, EndoActivator, sonic activation, EndoVac, and others were reported as effective devices to improve the irrigation [17]. Beside passive ultrasonic irrigation (PUI) which is a widely used technique [18], it improves mechanical NaOCl penetration [19], making it more effective over microorganisms [6] and organic tissues [20, 21]. Conversely, other studies show no further advantage of PUI activation over other techniques [22, 23].

This study aimed to evaluate the mechanical effect of PUI in dissolving organic tissue using 2.5% NaOCl and 2% CHX gel as endodontic irrigants. The null hypothesis tested was that the PUI activation would not improve the dissolving capacity of these irrigants.

## 2. Materials and Methods

This *in vitro* study was approved by the research ethics committee of São Paulo State University, Institute of Science and Technology (*n* 2.494.487). A total of 40 human lower premolars indicated for surgical extraction were obtained from patients undergoing orthodontic or periodontal treatment. Teeth were collected based on dimensional and morphological similarities which were evaluated by periapical radiography of all included teeth. Criteria such as the pulp chamber and root canal size, the presence of calcification, the presence of extra canals, endodontically treated teeth, and pulp calcifications were all evaluated while selecting the teeth to be included in this *in vitro* study.

### 2.1. Specimen Preparation

**Figure 1:**
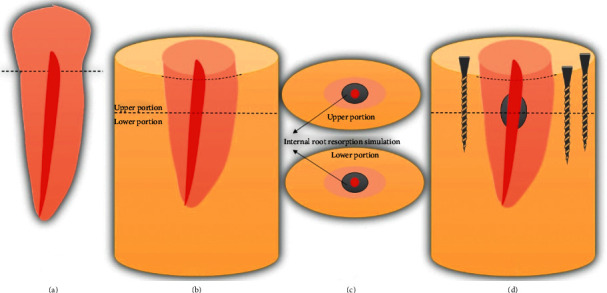
Schematic illustration of the IRR simulation. (a) The crown of each tooth was cross-sectioned at the cementoenamel junction, and (b) the roots were embedded in cylinders (3 cm diameter; 2.5 cm height) of self-cured acrylic resin. Then, (c) IRR was simulated, and (d) finally, the specimens were ready.

The crown of each tooth was cross-sectioned at the cementoenamel junction using a carborundum disk (Dentorium, New York, USA) under cooling, and the root length was standardized at 16 ± 0.5 mm. All roots were instrumented with K-file #15 (Dentsply Ind. Com. Ltda, Petrópolis, RJ, Brazil) and irrigated with 3 mL of 1% NaOCl. The roots were embedded in cylinders (3 cm diameter; 2.5 cm height) of self-cured acrylic resin (TDV, Santa Catarina, Brazil), and then an IRR was simulated [24] as shown in Figure 1 and detailed below.

With the aid of a dental parallelometer (Bio-Art, São Paulo, Brazil), the roots were fixed in cylinders (3 cm of diameter and 2.5 cm of height) of autopolymerized acrylic resin (TDV^®^, Santa Catarina State, Brazil) molded in silicone models (Silibor, Classico Artigos Odontológicos Ltda., São Paulo). In each resin block, 3 holes were made by using an electric drill with a bur of 3.96 mm of diameter, approximately 3 mm away from the root position and parallel to the long axis of the root, forming a triangle.

Using the IsoMet 1000 precision cutting machine (Buehler, Illinois, USA), the block was cross-sectioned at 8 mm far from its upper surface and perpendicularly to its long axis. Two portions, one upper and one lower, were obtained. Three cylindrical screws, 3.5 mm in diameter, were positioned in the holes and fixed with the aid of nuts to retake the original position of the block when necessary.

To simulate an internal resorption, a cavity of 1.25 mm of depth and 2.5 mm of diameter was prepared with diamond round bur 3030 (KG Sorensen, São Paulo, Brazil) on the inferior surface of the upper portion of the tooth sample and on the superior surface of the lower portion of the tooth sample (Figure 1). The upper and lower portions of the samples were repositioned with the screws.

### 2.2. The Organic Material

A tissue mass of porcine palatine mucosa was used to simulate the organic tissue [25] and adapted within the IRR simulation. It was weighed before the irrigation using an analytic balance (ATX UniBloc, Shimadzu, SP, Brazil); once the irrigation protocol was performed for each group (*n* = 10), the material was removed and dried using absorbing paper for 4 minutes in order to avoid humidity to affect the balancing process and finally weighed again; however, the mass remained partially wet.

### 2.3. The Experimental Groups

The total irrigation time was 150 s at a flow rate of 5 mL/min as the following:

- Group 1: CHX + PUI: the canals were filled with 1 mL of 2% CHX gel and irrigated with 5 mL of saline solution by typical syringe irrigation without PUI and then filled with 1 mL of 2% CHX gel and irrigated with 5 mL of saline solution activated with PUI. Finally, the canals were washed continually by 2.5 mL of ethylenediaminetetraacetic acid (EDTA) 17% for 30 seconds.
- Group 2: CHX: the canals were filled with 2 mL of 2% CHX gel and irrigated with 10 mL of saline solution by typical syringe irrigation without PUI activation and washed finally by 2.5 mL of ethylenediaminetetraacetic acid (EDTA) 17% for 30 seconds.
- Group 3: NaOCl + PUI: the canals were irrigated with 5 mL of 2.5% NaOCl by typical syringe irrigation using the needle 30G (NaviTip, Ultradent, South Jordan, UT, USA) without PUI and then 5 mL activated with PUI. Finally, the canals were washed continually by 2.5 mL of ethylenediaminetetraacetic acid (EDTA) 17% for 30 seconds.
- Group 4: NaOCl: the canals were irrigated with 10 mL of 2.5% NaOCl by typical syringe irrigation utilizing irrigation needle 30G without PUI activation and finally washed by 2.5 mL of ethylenediaminetetraacetic acid (EDTA) 17% for 30 seconds.

### 2.4. Passive Ultrasonic Irrigation

PUI activation was performed using an E1-Irrisonic stainless-steel tip (Helse, Santa Rosa de Viterbo, Brazil) at the working length using Soni^®^ II (Ortus, Paraná, Brazil) at 10% frequency. No movement was performed during PUI activation to avoid any contact between the ultrasonic tip and the canal walls.

### 2.5. Statistical Analysis

The difference between both readings (before and after irrigation) was calculated and transferred to percentage values. The obtained data were submitted to statistical analysis using two-way ANOVA (factors: irrigant type and activation methods: with/without PUI) and Tukey test for multiple comparisons among the experimental groups (*α* = 0.05).

## 3. Results

**Figure 2:**
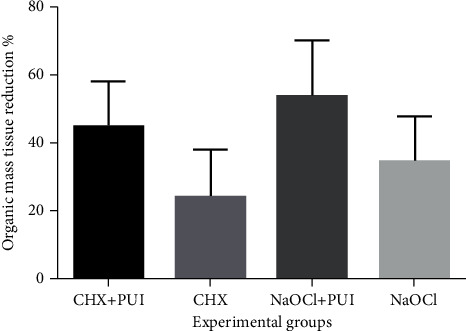
The statistical difference among the four experimental groups.

Tables 1 and 2 present the results of Tukey's test for the factors irrigant and PUI, respectively. There was a significant difference in both factors (irrigant: *p*=0.04; PUI: *p* ≤ 0.001). The groups that used PUI were more effective in dissolving the organic tissue of the IRR simulation than the groups without PUI as seen in Figure 2.

## 4. Discussion

The present study evaluated the effectivity of PUI in dissolving organic tissue when NaOCl and CHX were used. It can be noted that the mechanical effect of PUI was more evident than the chemical effect of irrigants over organic tissue dissolving (Table 2). The results showed that the groups of PUI activation were more effective over organic material than the groups that used conventional needle and syringe irrigation, and therefore, the null hypothesis was rejected.

Ultrasonic irrigation (UI) was introduced firstly by Richman to improve irrigants' action [26] in debris removal and organic tissue dissolution [27]. However, it is less effective than PUI due to reduced acoustic streaming [28]. Conversely, PUI has greater acoustic streaming and lower risk to cause iatrogenic accidents because it uses noncutting and smooth inserts [28–30].

In previous studies, PUI was effective in improving organic tissue dissolving due to the effect of the acoustic streaming and showed to be more effective than conventional needle and syringe irrigation [31]. Similar results were found in this study as PUI was more effective than conventional needle and syringe irrigation. The study of Al-Jadaa et al. [23] proved that the acoustic stream of PUI is more effective than the sonic stream in dissolving organic material, and this may be explained by its capacity to increase the irrigant temperature [32] or because it reduces the irrigant superficial tension playing the role of a chemical surfactant [25]. The results of the present study showed that PUI improves the organic tissue dissolution.

More recently, it was found that PUI is effective in reducing the organic material of narrow, infected, and curved root canals without the need for greater taper instruments; however, without the use of PUI, ≥35 taper instruments are indicated [33]. This factor was not evaluated in this *in vitro* study; however, it may be evaluated in future studies as minimally invasive endodontics is gaining a greater space.

NaOCl is an effective irrigant in dissolving organic material [34], and this agrees with other studies which showed similar results in bovine teeth but with 2.5% concentration [35, 36]. Similar results were found in the present study as 2.5% NaOCl was effective in organic tissue dissolution. However, the presence of EDTA as a final washing solution may reduce the dissolution capacity of NaOCl according to recent studies [37, 38].

CHX, as well, was studied to evaluate its dissolving capacity of organic tissues at a concentration of 2% [9]. In this study, the group CHX (without PUI) presented the lowest results with organic tissue dissolution (Figure 2 and Table 1) when compared with other groups, which is in agreement with other studies [39, 40]. Despite CHX presents long-lasting antimicrobial activity, as an endodontic irrigant, the lack of tissue dissolving capacity of CHX is a considerable drawback [41, 42].

Finally, it is important to mention that this *in vitro* study has some limitations as the balance process has an error margin because of humidity. Regardless the irrigant type, PUI was able to improve the dissolving capacity of the irrigant, and this is because of the acoustic streaming that maximizes the irrigant dissolving capacity over organic tissue. PUI was more effective than conventional needle and syringe irrigation, which agrees with other studies revealing the reduced effectivity of this method and the advantage of PUI over this method [31].

## 5. Conclusion

Passive ultrasonic irrigation is more effective than the conventional needle and syringe irrigation in organic tissue dissolution.

## Figures and Tables

**Table 1:** Tukey's test (*α* = 0.05) comparison of readings (means (%) and standard deviation (±SD)) for the isolated factor irrigant.

Type of irrigant	Means (±SD)
NaOCl	43.89 (±17.65)^a^
CHX	34.26 (±17.1)^b^

Mean values with different letters show a significant difference.

**Table 2:** Tukey's test (*α* = 0.05) comparison of readings (means (%) and standard deviation (±SD)) for the isolated factor PUI.

PUI	Means (±SD)
Without	29.21 (±14.33)^A^
With	48.95 (±15.56)^B^

Mean values with different letters show a significant difference.

## Data Availability

The data used to support the findings of this study are available at https://doi.org/10.7910/DVN/FFJD8A.
